# Spatially Resolved Biosensing of Localized Dopamine Release via Its Electropolymerization Using Plasmonic Electrochemical Microscopy

**DOI:** 10.3390/bios16050284

**Published:** 2026-05-14

**Authors:** Christian Martinez, Samuel Groysman, Madison Ngo, Yixian Wang

**Affiliations:** Department of Chemistry and Biochemistry, California State University, Los Angeles, Los Angeles, CA 90032, USA; cmart253@calstatela.edu (C.M.); sgroysm2@calstatela.edu (S.G.); mngo30@calstatela.edu (M.N.)

**Keywords:** dopamine, surface plasmon resonance, plasmonic electrochemical microscopy, polydopamine, electropolymerization, nanofilm, biosensing

## Abstract

The precise spatiotemporal monitoring of dopamine is critical for understanding neurotransmission and neurodegenerative pathologies. While traditional electrochemical methods offer excellent temporal resolution, they lack the spatial resolution required to map network-wide dynamic events. To address this, we adapted a wide-field plasmonic electrochemical microscopy (PEM) platform to spatially image localized electrochemical reactions. Specifically, we leveraged the anodic electropolymerization of dopamine into a surface-confined polydopamine nanofilm to enable label-free, pixel-level optical quantification. Bulk solution testing demonstrated highly uniform sensor sensitivity, yielding an estimated single-pixel limit of detection of 14 pM. Furthermore, utilizing a custom injection system, we successfully imaged the real-time localized delivery of micromolar dopamine concentrations and demonstrated qualitative responsiveness of the integrated optical signal to delivered dopamine as a proof-of-concept for the platform. The platform functions as a spatially resolved mass integrator while simultaneously decoupling this chemical signal from transient hydrodynamic mechanical deformations caused by dopamine injection flow. Ultimately, this platform establishes the fundamental methodology required for future high-throughput spatial monitoring of complex neurotransmitter release dynamics across cellular networks.

## 1. Introduction

Dopamine (DA) is an essential neurotransmitter in the nervous system that regulates motor function, spatial memory, and reward mechanisms [1,2,3]. Even subtle fluctuations in DA levels can lead to debilitating neurological disorders, including Parkinson’s disease, Huntington’s disease, and schizophrenia [4]. Importantly, DA signaling is a spatially and temporally heterogeneous process, with release and clearance varying across micrometer-scale regions and millisecond timescales [5,6,7,8]. Because extracellular DA concentrations are often low and fluctuate rapidly within localized domains, analytical tools must simultaneously achieve high sensitivity and spatiotemporal resolution to accurately capture these dynamic signals.

Electrochemical techniques are widely used for DA sensing because DA is electroactive and undergoes reversible redox reactions that can be readily detected at electrode surfaces [9]. Established approaches, most notably fast-scan cyclic voltammetry (FSCV), offer excellent sub-second temporal resolution and chemical selectivity [10,11,12]. Despite these strengths, FSCV typically relies on single-point measurements via carbon fiber microelectrodes, which lack the spatial resolution necessary to map the heterogeneity of DA release across cellular ensembles. While microelectrode arrays (MEAs) have been developed to expand spatial sampling by enabling parallel electrochemical measurements [13,14,15], they remain constrained by fabrication complexity, fixed electrode density, and potential signal interference between neighboring electrodes, which can hinder the resolution of fine-scale neurotransmitter dynamics.

Furthermore, sophisticated multimodal approaches have recently emerged, such as the coupling of FSCV with scanning ion conductance microscopy (SICM) [16]. This powerful combination enables high-resolution, topographically mapped chemical sensing with high temporal resolution. Yet, as a scanning probe technique, it is inherently limited by the trade-off between scanning speed and spatial area. There remains a significant need for wide-field imaging modalities that can capture simultaneous activity across the entire sample surface in real time. Additionally, optical sensing modalities, such as genetically encoded fluorescent sensors [17,18], non-genetically encoded nanosensors [19,20], and electrochemiluminescence imaging [21], offer impressive spatial imaging capabilities. However, these methods often require genetic modification or labeling that might potentially interfere with the underlying biological processes of interest.

Plasmonic electrochemical microscopy (PEM) emerges as a powerful, label-free alternative that addresses the technological hurdles outlined above. PEM is an imaging extension of surface plasmon resonance (SPR), a technique renowned for its high sensitivity in monitoring interfacial molecular processes in real time [22]. SPR-based sensors have already demonstrated robust detection of DA with high selectivity against common interferents like ascorbic and uric acid [23]. SPR has also been applied for measuring vesicle release from PC12 cells [24], but identifying chemicals in such living systems with SPR alone has not been reported. By coupling SPR with electrochemistry (EC-SPR), researchers have achieved picomolar sensitivity for DA through electrochemical polymerization into polydopamine (PDA) nanofilms [25,26]. While effective for bulk detection, these amplification strategies often lack the spatial resolution required for single-cell imaging. Other hybrid approaches have combined ultramicroelectrode sensors with SPR to monitor exocytosis [27,28], but electrochemical DA sensing remains localized to single-point measurements in these setups.

**Figure 1:**
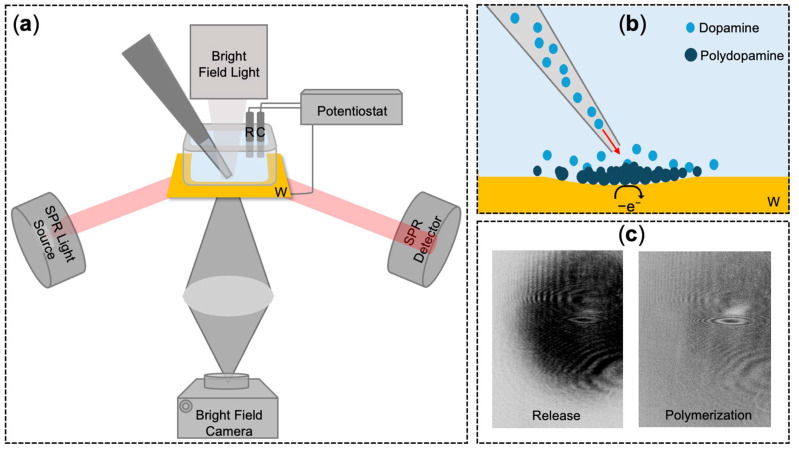
Experimental configuration and detection mechanism of the plasmonic electrochemical microscopy (PEM) system. (**a**) Schematic of the PEM setup integrated with an injection system. The three-electrode configuration utilizes a gold thin film as the working electrode (W), with an Ag/AgCl reference electrode (R) and a platinum wire counter electrode (C) controlled by a potentiostat. (**b**) Detailed view of the sensing mechanism: a micropipette provides a confined release of dopamine (DA), which undergoes electrochemical oxidation at the gold surface to form a localized polydopamine (PDA) film. The schematic also illustrates the transient mechanical deformation (local dip) on the sensor surface caused by the hydrodynamic pressure of the pumped solution flow. (**c**) Representative PEM images highlighting the distinction between physical and chemical signals: the transient mechanical impact during initial release shown as the dark pattern (left) and the persistent bright signal generated by dopamine polymerization (right).

By integrating an optical microscope into an EC-SPR system, PEM enables real-time mapping of local surface activity. This configuration allows for the visualization of optical changes induced by localized electrochemical reactions across a wide field of view (Figure 1) [29,30,31]. Compared to established optical and electrochemical methods, PEM offers a unique combination of advantages: it is label-free, highly surface-sensitive due to the SPR evanescent field, and achieves chemical specificity through the electrochemical reaction itself. In this configuration, the gold SPR film serves as both the plasmonic sensor and the working electrode (W) in a standard three-electrode electrochemical cell. Our laboratory has previously utilized PEM for imaging heterogeneous electrochemical responses in systems such as gold nanoparticles, Prussian blue nanoparticles, and nickel hexacyanoferrate (NiHCF) thin films [32,33,34]. We also recently demonstrated PEM as a spatially resolved biosensor for hydrogen peroxide [35] and dopamine [36], achieving sensitivity enhancement through coating the gold sensor chip with a Prussian blue thin film or mesoporous silica film, respectively.

In this work, we significantly advance these capabilities by implementing a PEM protocol that leverages localized dopamine polymerization to provide high-contrast, high-sensitivity mapping of DA release sites (Figure 1). We first build upon an established polymerization protocol [26] to demonstrate our imaging system’s sensitivity toward bulk dopamine solutions. To model localized signaling, we designed an injection system using a micropipette to enable the confined release of analyte. Through this setup, we also characterized the system’s responsiveness to the hydrodynamic pressure of the pumped solution flow, establishing a baseline for distinguishing chemical signals from physical disturbances. Finally, the platform was applied to the real-time mapping of dopamine release at varying amounts, where the integrated optical signal demonstrated qualitative responsiveness to the nominal amount of dopamine delivered as a proof-of-concept for spatially resolved mass sensing. This multifaceted approach positions PEM as an efficient and powerful platform for mapping of integrated diffusion profiles across the entire field of view and potential high-contrast identification of secretory regions across cellular networks.

## 2. Materials and Methods

### 2.1. Chemicals and Materials

6-mercaptohexanoic acid (MHA) was purchased from Sigma-Aldrich (St. Louis, MO, USA). 2-mercaptoethanol (ME) was purchased from Matheson Coleman & Bell (Los Angeles, CA, USA). Dopamine hydrochloride (DA, 99%) was purchased from Alfa Aesar (Ward Hill, MA, USA). Ethanol (anhydrous), hydrochloric acid (HCl, 37%), and phosphate-buffered saline (PBS) tablets were purchased from Thermo Fisher Scientific (Waltham, MA, USA). All chemical reagents were analytical grade and used without further purification. All aqueous solutions were prepared using double-deionized water (dH_2_O, resistivity = 18.2 MΩ·cm at 25 °C, Milli-Q Ultrapure water EQ 7000 Purification System, MilliporeSigma, Burlington, VT, USA). Gold (Au) sensor chips (50 nm Au on 2 nm Ti) were purchased from Biosensing Instruments (Tempe, AZ, USA). Ag/AgCl reference electrodes and Pt wire counter electrodes were purchased from CH Instruments (Austin, TX, USA). Silicon wells for the electrochemical cell were cut from flexiPERM slides (Sarstedt, Nümbrecht, Germany).

### 2.2. Modification of Au Sensor Chips

The Au sensor chips were modified following reported protocols [26]. Prior to modification, the Au sensor chips were rinsed sequentially with ethanol and dH_2_O (3×) and then briefly annealed with a hydrogen flame. The cleaned electrodes were incubated overnight at 4 °C in an ethanol solution containing 10 µM MHA. Following incubation, the electrodes were rinsed sequentially with ethanol and dH_2_O, then immersed in 1 µM 2-mercaptoethanol for 30 s. Finally, the chips were rinsed and dried under a stream of nitrogen.

PBS (1×) was prepared by dissolving one tablet into 200 mL of dH_2_O and adjusting the pH to 6.8. This buffer was used for the preparation and dilution of all experimental solutions. A 0.1 M DA stock solution was prepared in PBS and used to generate a series of 10-fold serial dilutions. To ensure purity and remove particulates, all experimental solutions were filtered through a 0.2 µm syringe filter (Global Life Sciences Solutions Operations UK Ltd., Buckinghamshire, UK).

### 2.3. Electrochemical Cell and PEM Setup

All electrochemical measurements were monitored in situ using a PEM system. This platform couples an SPR microscope (SPRM 200 Series, Biosensing Instruments) to an electrochemical cell governed by a CHI 760E potentiostat (CH Instruments). The potentiostat controls the electrochemical reactions within a standard three-electrode configuration, utilizing the Au chip as the working electrode (with an exposed geometric area of 0.9 cm^2^), a Pt wire counter electrode, and an Ag/AgCl reference electrode. To enable plasmonic imaging, p-polarized light from a 690 nm laser source was directed onto the backside of the Au chip through a prism to induce surface plasmon excitations, and variations in the reflected light intensity were captured by an SPR detector. The incident angle for imaging was fixed slightly below the surface plasmon resonance angle. With a field of view of 450 × 600 µm^2^ mapped to a 480 × 640 pixel CCD array, the system provides a digital sampling resolution of ~0.94 μm per pixel. The experimental optical resolution was verified using 10 µm polystyrene microbead standards (Sigma-Aldrich) and gridded sensors, demonstrating a resolution of ~2 µm perpendicular to the direction of plasmon propagation and tens of microns along the direction (see Section S1 and Figures S1 and S2 in the Supporting Information). PEM image stacks were acquired at either 1 or 50 frames per second (fps) using Image SPR 2.4 software (Biosensing Instruments).

### 2.4. PEM Measurements of Bulk and Localized Dopamine Injections

For bulk injection experiments, chronoamperometry was performed at a constant applied potential of +0.2 V. Following a 2-min equilibration period to establish a stable baseline, 4 µL of the first DA stock solution (10 nM) was introduced into the 400 µL buffer to reach a bulk concentration of 0.1 nM and monitored for 10 min to capture the polymerization process. This sequence was performed sequentially for all other stock solutions (100 nM, 1 µM, 10 µM, and 100 µM), reaching final concentrations of 1 nM, 10 nM, 100 nM, and 1 µM, respectively. Finally, a control injection of 1% ethanol was applied to calibrate the optical signal to standard units. This 1% ethanol increase corresponds to a theoretical uniform shift of 60 mDeg [37].

For localized delivery experiments, DA was dispensed from a quartz micropipette (approximate tip diameter of 10 μm) positioned directly above the modified Au sensing surface. The micropipettes were fabricated from quartz capillaries (1.0 mm outer diameter, 0.5 mm inner diameter, Sutter Instrument, Novato, CA, USA) using a P-2000 laser puller (Sutter Instrument). The approach of the pipette was precisely controlled using a 3-axis motorized micromanipulator system (TRIO/MP-245A, Sutter Instrument) integrated with a CV 203BU headstage (Molecular Devices, San Jose, CA, USA). After filling the micropipette with the designated DA concentration, it was advanced toward the substrate under continuous live PEM monitoring. A localized optical response served as an indicator of surface proximity, allowing the tip to be accurately positioned above the target sensing area. Local DA delivery was subsequently actuated by applying positive air pressure through a syringe pump coupled to the back of the micropipette, while maintaining a continuous electrochemical bias of +0.2 V throughout the duration of the measurement.

### 2.5. Data Analysis

#### 2.5.1. General Processing Protocols

Raw PEM image stacks were converted into .tif image sequences using ImageAnalysis software (Biosensing Instruments, 2.4) and subsequently imported into ImageJ 1.53t (National Institutes of Health, Bethesda, MD, USA) for formatting. To enhance contrast and isolate dynamic interfacial events, background subtraction was applied to the majority of the image sets. Subsequent spatial and temporal analyses were executed using either ImageJ or MATLAB (R2025b, MathWorks, Natick, MA, USA).

#### 2.5.2. Pixel-Wise Analysis of Concentration Dependence for Bulk Injection

PEM images acquired at the 10-min endpoint of each bulk injection were analyzed utilizing a custom MATLAB script. To mitigate high-frequency spatial noise, the raw images were initially subjected to a 5 × 5 spatial averaging filter. The optical intensity of each pixel was then converted to standard resonance angle shift units (mDeg) by applying a pixel-specific calibration matrix derived from the 1% ethanol control injection, unless otherwise noted. Notably, this pixel-by-pixel multiplication inherently corrects for uneven optical illumination across the sensing area. Following this calibration, the localized optical response was correlated with the corresponding bulk dopamine concentration. A linear regression was executed independently for every pixel across the image stack to extract the calibration slope and the coefficient of determination (R^2^). Finally, these extracted metrics were reconstructed into heatmaps to map the spatial distribution of sensor sensitivity and linearity.

#### 2.5.3. Image Analysis of Local Delivery Data

Image sequences capturing localized delivery events were background-subtracted in ImageJ using the frame immediately preceding the injection event as a reference baseline. Spatial line profiles corresponding to distinct temporal points were extracted and subsequently analyzed using MATLAB. The raw intensity profiles were smoothed using a moving average filter with a 10-point window. The filtered data were then plotted as stacked, top-down temporal profiles. To accurately convey the true signal magnitude despite the vertical offsets required for stacking, each plotted profile was dynamically color-mapped according to its original, un-offset intensity values.

Google Gemini 3 was used to draft the MATLAB code. The complete MATLAB codes are available at our GitHub repository (https://github.com/ywang184/MATLAB_DopamineSensing, accessed on 13 May 2026).

## 3. Results

### 3.1. PEM Measurement of Bulk Dopamine Injection

**Figure 2:**
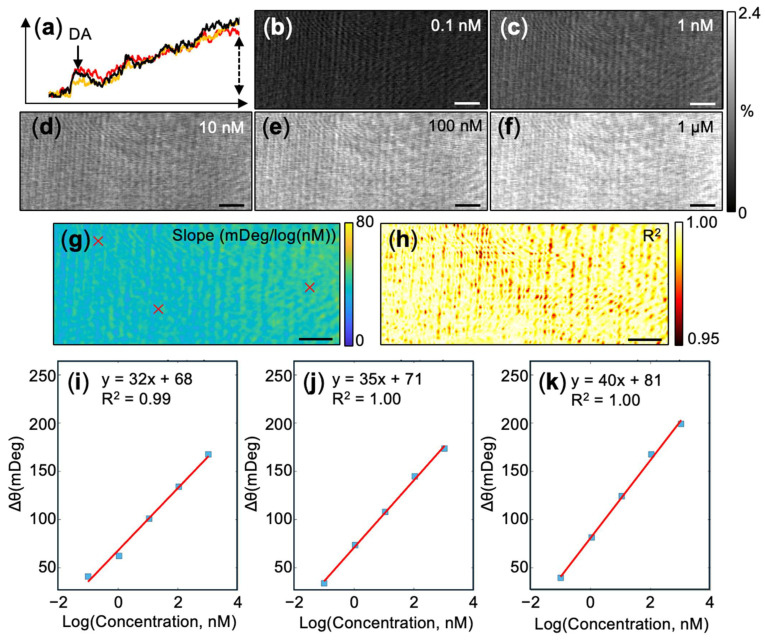
Spatially resolved PEM imaging of bulk dopamine polymerization. (**a**) Real-time temporal intensity profiles extracted from three randomly selected pixels, showing the continuous optical response following the injection of 4 µL of 10 nM DA into 400 µL of PBS buffer (the injection frame is indicated by the arrow). (**b**–**f**) Representative background-subtracted PEM images captured at the 10-min endpoint of sequential bulk dopamine injections from 0.1 nM to 1 µM. The grayscale bar indicates the percentage change in reflected light intensity. (**g**) Pixel-wise heatmap displaying the spatial distribution of the calibration slope (mDeg/log(nM)). (**h**) Corresponding spatial heatmap of the coefficient of determination (R^2^). (**i**–**k**) Linear calibration plots extracted from the three pixels marked by red crosses in panel (**g**). The scale bar is 50 µm in panels (**b**–**h**).

To establish the quantitative mapping capabilities of the PEM platform, we first evaluated its response to bulk dopamine injections. The detection mechanism relies on the electropolymerization of dopamine at a constant anodic potential (+0.2 V) following its electrostatic accumulation on the MHA-modified gold surface [26]. The continuous deposition of PDA induces a cumulative local refractive index change, enabling picomolar sensitivity [26]. Using the PEM setup, increasing concentrations of dopamine were sequentially injected into a PBS solution to reach bulk concentrations from 0.1 nM to 1 µM, allowing the interfacial polymerization to proceed for 10 min prior to each subsequent injection. Figure 2a illustrates the real-time optical intensity profiles extracted from three individual spatial coordinates (pixels, approximately 1 μm × 1 μm) during the first injection. Following a transient optical spike caused by the injection perturbation, the signal exhibits a continuous increase indicative of localized polymer nanofilm growth (profiles were smoothed using a 5-point moving average).

The spatial uniformity of this signal change across the sensor surface was visualized by subtracting the pre-injection baseline from the 10-min endpoint frame for each concentration, revealing a clear, dose-dependent contrast enhancement (Figure 2b–f). The presence of residual optical interference fringes, visible as subtle ribbon-like patterns in the spatial heatmaps, is likely attributable to coherent scattering or minor optical aberrations inherent to the aged optical path of the instrument. While it is difficult to remove computationally, potential mitigation strategies are outlined in the Discussion (Section 4.1). To rigorously quantify this response, a pixel-wise linear regression was applied across the tested concentration window. The raw PEM intensity changes were calibrated to standard resonance angle shifts (mDeg) using a 1% ethanol control injection, which corresponds to a 60 mDeg shift [37]. This generated high-resolution spatial heatmaps of the calibration slope (Figure 2g) and the coefficient of determination, R^2^ (Figure 2h). The slope heatmap mapped the localized sensitivity across the sensor interface, while the R^2^ heatmap confirmed the high linearity of the sensor response at the micro-scale. Representative calibration plots extracted from three designated pixels (Figure 2i–k) demonstrate robust, quantitative detection at the single-pixel level. Overall, the imaging platform proved highly uniform and sensitive, exhibiting an average slope of 40.0 ± 3.5 mDeg/log(nM) and R^2^ values consistently approaching 1.0 across the active area. Global calibration equations derived from this experiment and two independent replicates were y = 40x + 79, y = 32x + 82, and y = 38x + 127. For the latter two replicate experiments, elevated optical noise during the ethanol control injections precluded the use of a pixel-by-pixel normalization matrix. Instead, a globally averaged ethanol calibration factor was calculated and applied uniformly across all pixels.

Finally, we estimated the single-pixel limit of detection (LOD) using the baseline noise variation against the global calibration curve. Specifically, the LOD was calculated using the following equation:

$$LOD=10^{\left(3.3\sigma -intercept\right)/slope}$$

where σ is the standard deviation of the per-pixel optical intensity measured across a 200-s pre-injection baseline window, sampled at 1 frame per second (i.e., *n* = 200). The σ value was computed independently for three random pixels. The intercept and slope were taken from the calibration equation y = 40x + 79 from the same sensor. This yielded an estimated LOD of 10^−1.85±0.03^ nM (≈14 pM). While this is an order of magnitude higher than the bulk 1.4 pM LOD previously reported for non-imaging EC-SPR systems [26], this variance is entirely anticipated for single-pixel measurements.

### 3.2. PEM Imaging of Localized Dopamine Delivery

#### 3.2.1. Local Delivery Setup and Visualization

**Figure 3:**
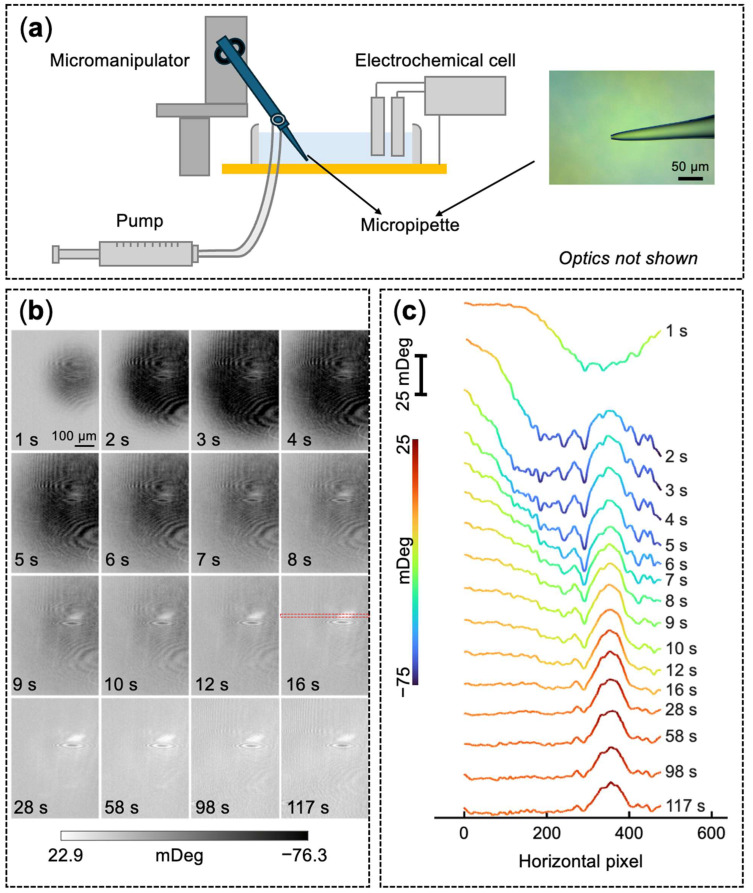
Real-time spatially resolved PEM imaging of localized dopamine delivery and polymerization. (**a**) Schematic of the localized delivery setup, showing a quartz micropipette positioned by a micromanipulator and pressurized by a syringe pump. The inset displays an optical micrograph of the micropipette tip. (**b**) Time-lapse sequence of background-subtracted PEM images showing the dynamic surface response. (**c**) Stacked spatial intensity profiles at sequential timepoints, extracted across the deposition site (marked by the red dashed rectangle in panel (**b**) at 16 s). For these measurements, a 10 µm micropipette prefilled with 1 µM DA in PBS was positioned approximately 10 µm above the sensor interface, and localized delivery was actuated via positive air pressure from a 10 mL syringe.

To simulate the localized, transient nature of endogenous DA release from cellular vesicular exocytosis, we assembled a microinjection delivery system. As depicted in Figure 3a, a quartz micropipette (~10 µm tip diameter) prefilled with 1 µM DA was precisely positioned using a micromanipulator. Positive air pressure was applied via a syringe pump to actively drive the discrete extrusion of the DA solution over a brief period.

The time-lapse PEM sequence (Figure 3b, Video S1) captures the dynamic interfacial response during and immediately following this injection phase. During the active delivery stage (1 to 4 s), a rapidly expanding dark region (negative angular shift) appeared on the sensor surface. We attribute this to the transient mechanical deformation caused by the hydrodynamic flow of the injection, based on a similar observation in a pure PBS injection (Figure S3). After the pump was stopped and pressure was released, this dark circular footprint gradually dissipated. Concurrently, a highly localized bright spot emerged at the center of the injection zone and persisted throughout the remainder of the measurement window, indicating a positive angular shift caused by the oxidative conversion of DA to PDA. Notably, the spatial footprint of this primary bright spot did not increase substantially over time. A slightly elevated signal was observed gradually developing in the region immediately surrounding the primary deposition site, corresponding to the radial growth of the PDA film. Similarly to Figure 2, residual optical imaging artifacts are present, and they manifest as unchanging scattering patterns across all images. While not detrimental to image analysis, these can be mitigated in future experiments (see Discussion, Section 4.1). The dynamically color-mapped cross-sectional profiles (Figure 3c) trace the temporal evolution, capturing the initial negative pressure-induced dip transitioning directly into the coherent, growing positive peak.

#### 3.2.2. Concentration and Volume Dependence of Localized Dopamine Delivery

**Figure 4:**
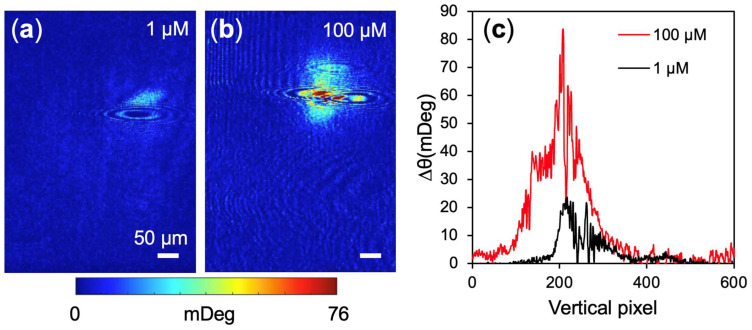
PEM response to localized dopamine delivery at varying concentrations. (**a**,**b**) PEM images mapping the spatial distribution of the electropolymerized polydopamine film following the injection of (**a**) 1 µM and (**b**) 100 µM dopamine solutions. (**a**) was collected 45 s after injection and (**b**) was 24 s after injection. (**c**) Vertical cross-sectional intensity profiles extracted across the center of the respective polymerization sites.

To validate the quantitative mapping capabilities of the platform, we tested the concentration dependence of the localized response. When delivering a 100-fold higher concentration (100 µM), a larger and more intense bright deposition pattern was observed on the sensor surface (Figure 4a,b). The cross-sectional profiles in Figure 4c demonstrate a concentration-dependent increase in both the maximum optical shift (peak height) and the vertical spatial spread of the deposited PDA film. The fluctuations and noise observed in the line profiles are due to optical pixel noise, residual imaging artifacts, and the presence of impurities that scatter the surface plasmon polaritons. This scattering generates the distinct parabolic tails clearly visible in the corresponding images. Notably, these parabolic interference patterns could also originate from isolated, nanoscale PDA structures rather than external impurities, indicating an inherently heterogeneous polymerization process (see Section 4.1).

**Figure 5:**
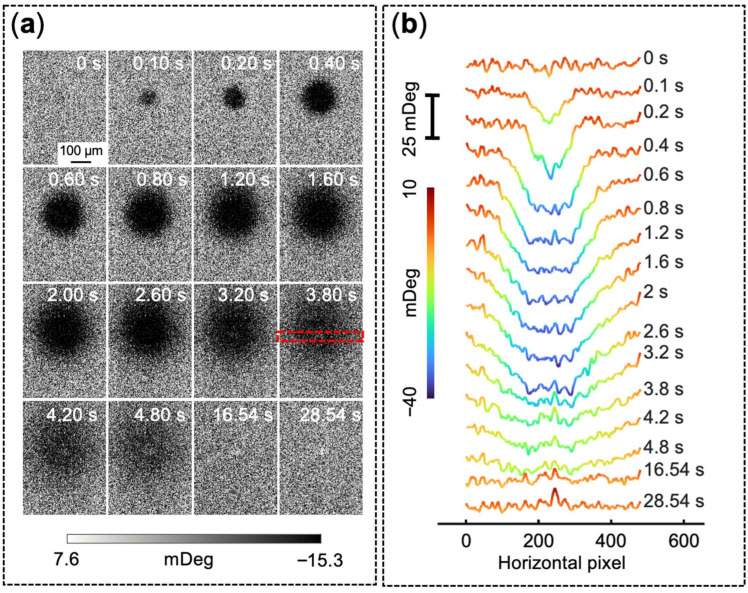
Real-time spatially resolved PEM imaging of localized dopamine delivery utilizing a lower-pressure 1 mL syringe. (**a**) Time-lapse sequence of background-subtracted PEM images capturing the dynamic surface response. (**b**) Stacked horizontal spatial intensity profiles extracted across the delivery site (marked by the red dashed rectangle in panel (**a**) at 3.80 s).

We subsequently evaluated the sensor’s dependence on the total volume of fluid delivered by substituting the 10 mL syringe with a 1 mL syringe, thereby reducing the applied driving force. As shown in the time-lapse sequence in Figure 5a, a much smaller dark mechanical deformation pattern was observed during the injection phase compared to Figure 3b, consistent with the reduced hydrodynamic flow. Following the dissipation of this pressure-induced dip, the stacked spatial profiles (Figure 5b) reveal a rapid baseline recovery with a much less pronounced positive angular shift compared to the initial higher-pressure test. While a bright deposition spot is still visible, it is significantly less intense, and its horizontal spatial footprint is reduced to approximately half of that observed with the 10 mL syringe (~50 µm versus 100 µm, as detailed in Figure 3c). The magnitude of the signal, which is directly proportional to the localized film thickness [32], varied substantially. By integrating the total pixel intensity within the polymerized film region, we calculated a total integrated signal of 59,777 a.u. for the 10 mL syringe delivery and 4991 a.u. for the 1 mL syringe delivery. This approximate 12-fold difference in the integrated optical signal aligns closely with the anticipated order-of-magnitude reduction in delivered volume, demonstrating that the integrated signal scales with delivered mass. It should be noted that because the optical signal from the 1 mL syringe delivery approaches the baseline background noise, the integration value carries higher uncertainty; nevertheless, the proportional scale of the mass reduction remains distinct.

#### 3.2.3. Mass-Responsive Imaging of Localized Dopamine Delivery

**Figure 6:**
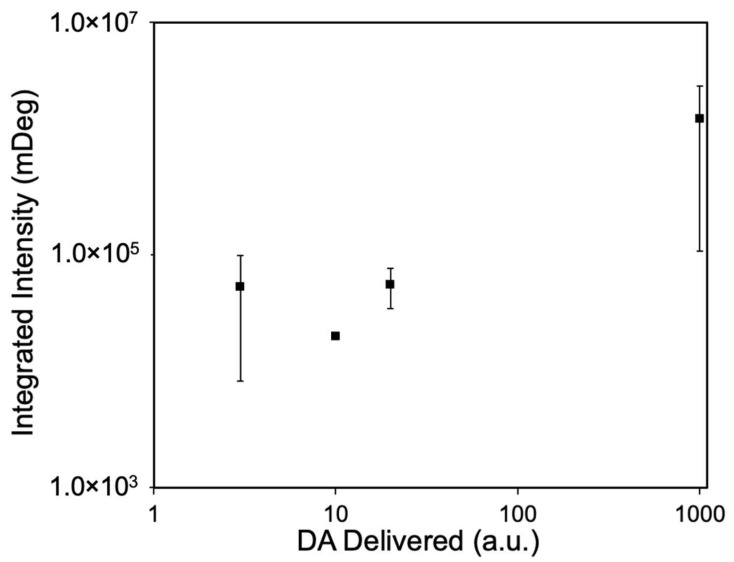
Mass-responsive optical signal of the PEM platform across delivered DA amounts. The plot presents the relationship between the integrated plasmonic intensity and the nominal amount of DA delivered on log–log axes to visualize the response across a wide dynamic range. Error bars represent the standard deviation across replicate injections. Data points represent nine injections across four combinations of DA concentration and pump-displaced volume (1 μM × 3 mL, *n* = 3; 10 μM × 1 mL, *n* = 1; 10 μM × 2 mL, *n* = 3; 100 μM × 10 mL, *n* = 2).

To evaluate the responsiveness of the platform to varying amounts of delivered dopamine, we performed independent injections using varying combinations of DA concentrations and delivery volumes. The resulting integrated intensity was plotted against the nominal amount of DA delivered, shown in Figure 6. Since the direct measurement of the absolute volume reaching the sensor interface is hindered by fluidic resistance and system dead volume, we estimated the nominal amount delivered using the product of the DA concentration (μM) and the volume displaced by the syringe pump (mL). We acknowledge that the actual volume reaching the sensor surface is distinct from the volume pushed through the syringe. However, because these two values are assumed to be proportional within the experimental regime, this proxy allows for a reliable quantitative scaling. Accordingly, the calibration values are presented in arbitrary units (a.u.) to maintain a robust correlation without asserting an absolute mass accuracy that the current delivery system cannot independently verify. To ensure analytical consistency across diverse deposition morphologies, we utilized a fixed-size region of interest (ROI, circular, 166 μm diameter, large enough to cover most of the primary deposition patterns) for signal integration across all injections. While this approach may slightly underestimate the total signal across the entire sensor surface, it represents the most robust solution for excluding the noise in the presence of background interference fringes and scattering patterns.

The data is presented on a log–log axis to effectively visualize the results across multiple orders of magnitude. Error bars span up to roughly an order of magnitude at some conditions, reflecting substantial variability in integrated signal at nominally identical delivered amounts. We attribute this variability primarily to the current injection system’s reliance on manually actuated syringe pressure, which limits the precision of delivered volume from the micropipette. Given this variability, particularly at the extremes of the tested range, the data should be interpreted as a qualitative proof-of-concept for mass-responsive optical imaging rather than as a validated quantitative calibration. The overall trend across three orders of magnitude nevertheless supports the mass-integration behavior of the platform and motivates the development of a next-generation injection system with improved volume control, as discussed in Section 4.2.4.

## 4. Discussion

### 4.1. Spatially Resolved Imaging and Real-Time Decoupling of Physical and Chemical Interfacial Phenomena

The localized delivery experiments successfully demonstrate the unique capability of the PEM platform to spatially monitor DA release and simultaneously resolve and decouple concurrent physical and chemical interfacial phenomena in real time. The ability to image transient mechanical deformation provides an invaluable internal reference. It offers a potential mechanism to optically estimate or normalize the specific volume of fluid delivered during a discrete, micro-scale release event by quantifying the magnitude and spatial spread of the pressure dip.

Following the pressure cessation, the subsequent persistent positive angular shift indicates the localized formation of the PDA film. The observation that the primary deposition spot did not expand substantially over time suggests that the highly localized sensing region rapidly reached surface saturation, and/or the finite mass of delivered dopamine quickly diffused away from the central release site. This diffusion dynamic is further supported by the emergence of the slightly elevated optical signal surrounding the primary injection zone, which successfully maps the radial expansion and subsequent low-concentration polymerization of the unconfined dopamine across the wider sensor interface.

Crucially, while the hydrodynamic mechanical deformation presented as a highly symmetric, circular footprint in both Figure 3 and Figure 5, the subsequent primary PDA deposition pattern exhibited distinct spatial asymmetry. This morphological divergence supports the conclusion that the persistent optical signal originates strictly from the localized surface product (the polymerized nanofilm) rather than a temporary refractive index gradient of the injected dopamine solution. Furthermore, as noted in Figure 4a,b, distinct parabolic interference tails were observed, which are characteristic of surface plasmon polariton scattering by nanostructures [31]. In addition to potential ambient contamination, these tails likely originate in part from isolated PDA nanostructures. Together, these observations highlight the inherently heterogeneous nature of surface-confined electropolymerization and suggest it follows a localized nucleation and growth mechanism that is highly sensitive to its immediate microscopic environment. Consequently, the observed optical scattering effectively maps the heterogeneous kinetics of surface-confined polymerization, providing morphological insight that traditional single-point electrochemical techniques cannot resolve. Because the micropipette was approached at a non-vertical angle, the lateral mass transfer component introduced by the direction of the injection could also contribute to the geometry of the deposition pattern. Nevertheless, while this inherent sensitivity is analytically powerful, transitioning to live-cell studies will require careful separation of signal heterogeneity arising from intrinsic sensor and polymerization variability versus heterogeneity from actual biological release patterns.

For a quantitative wide-field imaging sensor, establishing absolute surface uniformity and maintaining an interference-free background are critical prerequisites. In our current work, background-subtracted images exhibit subtle fringe patterns and scattering artifacts. These features are likely attributable to the degradation of the PEM system’s optical components, specifically minor scratches on the prism surface and long-term drift in the optical alignment. We anticipate that these localized background issues can be addressed in future iterations by replacing the aging optics and implementing advanced fringe-removal modules. Beyond hardware optimization, image processing strategies can be employed to further enhance the signal-to-noise ratio and spatial fidelity, such as calculating the temporal derivative of the image sequence instead of relying on a static first-frame subtraction to efficiently suppress background fringes and patterns caused by slow system drift, with the tradeoff of reduced sensitivity to slowly developing signals.

### 4.2. Evaluation of Analytical Performance

#### 4.2.1. Sensitivity and Limit of Detection

Our bulk dopamine characterization establishes the platform’s robust capacity for pixel-level, spatially resolved quantification of dopamine with a limit of detection comparable to traditional, non-imaging EC-SPR measurements, while fundamentally overcoming the spatial limitations of those prior methodologies. If desired, the effective LOD can be improved through additional smoothing in both temporal and spatial domains. This dual capability of maintaining high sensitivity while adding a spatial dimension is a notable analytical advance. On the other hand, achieving low nanomolar sensitivity during discrete local delivery events remains a challenge due to the finite absolute mass of dopamine released. To capture the analyte more efficiently, further interfacial modifications are required. Future work will systematically evaluate sensitivity-enhancing capture strategies, such as the integration of mesoporous silica films [38], to maximize the localized accumulation of dopamine prior to polymerization. Beyond interfacial capture strategies, future iterations of the platform could also benefit from nanostructured plasmonic architectures that enhance the intrinsic refractive-index sensitivity of the sensor surface itself [39,40,41]. Combining such structural sensitivity enhancements with the electrochemical amplification and wide-field imaging capabilities demonstrated here represents a promising direction for improving single-pixel detection limits during localized delivery events.

#### 4.2.2. Spatial and Temporal Resolution

The system provides a digital sampling resolution of 0.94 μm per pixel and an optical resolution of ~2 μm perpendicular to the plasmon propagation direction. The latter could be enhanced by transitioning to a home-built, objective-based PEM configuration, which could achieve diffraction-limited, nanometer-scale resolution, potentially augmented further by computational post-processing techniques [31,42]. The optical resolution along the propagation direction is constrained by the intrinsic plasmon propagation length, which can be improved through specialized hardware and software strategies such as adoption of line-scan imaging modes [43], multiangle incident light modulation [44], or mathematical image reconstruction algorithms [45]. Despite these intrinsic constraints, the current prism-based configuration offers a distinct and highly practical advantage: a macroscopic wide field of view (600 × 450 µm^2^). This expansive imaging area is exceptionally well-suited for monitoring heterogeneous chemical gradients across large cellular populations, where mapping network-level activity is often more informative than single-synapse interrogation. Furthermore, because extracellular dopamine rapidly diffuses away from localized release sites, capturing ultra-high nanoscale resolution is not strictly necessary to accurately monitor physiologically relevant spatiotemporal release dynamics.

Mass transfer dynamics dictate the ultimate spatial resolution of the system. The active pumping mechanism utilized with the 10 µm micropipette introduces a strong convective flow, causing the deposition footprint to span hundreds of micrometers. In physiological in vitro models, such as mapping exocytosis in PC12 cell networks, the endogenous release mechanism produces far smaller volumes, meaning convective spreading will be minimized. Nevertheless, radial diffusion will always be present. Achieving true cellular (tens of micrometers) or subcellular (micrometer) spatial resolution will require minimizing the separation distance between the biological sample and the plasmonic sensor. We are currently designing a next-generation PEM system optimized for real-time live-cell studies, featuring precise, micrometer-scale control over this separation distance (see Section 4.4).

The temporal resolution of the current system is limited to the minute timescale for nM bulk measurements and the second timescale for µM local delivery, which make it unsuitable for resolving ultrafast, sequential exocytotic events occurring at the exact same spatial coordinate. Instead, it functions as a mass integrator, which is discussed in detail in Section 4.3. Nevertheless, improving the interfacial capture strategy will significantly decrease the sampling time required to reach a viable signal-to-noise threshold. Furthermore, coupling the PEM platform with rapid, reversible detection modalities such as FSCV will be explored, with careful sensor design to account for capacitive charging currents.

#### 4.2.3. Specificity and Selectivity

The analytical selectivity of the PEM platform toward DA is a critical factor for its eventual application in complex biological environments. In this proof-of-concept study, we focused on establishing a framework for spatially resolved mass sensing and did not perform systematic interference testing against common physiological interferents, such as ascorbic acid (AA) and uric acid (UA), or dopamine analogues, such as norepinephrine, epinephrine, L-DOPA, and serotonin. Instead, our approach relies on the well-documented electrochemical behavior of DA reported in the literature. Selectivity against AA and UA under the protocol applied here has been experimentally demonstrated [26]. This chemical specificity can be explained by a threefold selectivity mechanism: AA and UA are unable to undergo electropolymerization, they are excluded from the negatively charged surface, and they are less readily oxidized than DA at +0.2 V. Dopamine analogues, on the other hand, could potentially undergo similar electropolymerization processes [46,47,48]. It has been reported that the number of hydroxyl groups on the benzene ring and the type of amine group (primary versus secondary) significantly affect electropolymerization kinetics [49]. For example, L-DOPA has a slower electropolymerization rate compared to DA, which may contribute to the chemical specificity of our system. Future work will rigorously evaluate the platform’s selectivity against common electroactive interferents and within complex media. The inherent selectivity could potentially be enhanced through interfacial engineering, such as by applying ion-selective membranes like Nafion to preclude neutral and anionic interferents [50], utilizing size-exclusion layers like mesoporous silica films, or integrating specific receptors such as molecularly imprinted polymers [51].

#### 4.2.4. Reproducibility

Bulk dopamine characterization through three independent PEM sensor chips yielded global calibration equation slopes of 40, 32, and 38 mDeg/log(nM), demonstrating a reproducible concentration-dependent sensitivity. The slight variation (11% relative standard deviation) observed across these replicates is primarily attributed to differences in the density and structural organization of the MHA self-assembled monolayer formed during sensor preparation. A moderate variation (28% relative standard deviation) was observed in the y-intercepts of the global equations (79, 82, and 127 mDeg, respectively). This likely reflects the day-to-day variations in the absolute baseline that reflect minor macroscopic differences in the optical coupling efficiency at the prism interface and the exact incident angle selected during system initialization. Nevertheless, calibrating the sensor platform during each individual use is highly recommended to ensure maximum quantitative accuracy.

The local injection experiments were accompanied by a higher variance than the bulk detection experiments. The large standard error at each delivery point reflects substantial injection-to-injection variability. As mentioned in Section 3.2.3, this is primarily attributed to the limitations of the manual injection system, where precise control over the delivered volumes is difficult to maintain consistently. Other factors, including pipette–sensor distance and approach angle, may also contribute. To enhance reproducibility in future iterations, we will precisely monitor the injection angle and transition to automated microinjection or iontophoresis, which provides fast and precise control of DA release [52].

### 4.3. PEM as a Spatially Resolved Mass Integrator and Its Biological Relevance

The spatially resolved signal in the PEM platform is generated by the electrochemical polymerization of DA into a surface-confined PDA film. Consequently, the observed optical patterns reflect the cumulative deposition of polymer at each pixel rather than the instantaneous concentration field of DA in the surrounding solution. This distinction is critical, as the platform functions as a spatially resolved mass integrator rather than a real-time concentration imager. Within this framework, PEM offers three capabilities beyond what bulk electrochemistry and single-point measurement provide: the spatial mapping of released and captured DA across a wide field of view, the integration of accumulated signal at each pixel reflecting locally captured mass, and the optical decoupling of persistent chemical signals from transient hydrodynamic deformations.

This integrator modality matches the neurobiological systems it is designed to interrogate. Extracellular dopamine signaling operates primarily through volume transmission, where neurochemical flux is organized into micrometer-scale domains rather than uniform, diffuse clouds [6,53]. In the striatum, functional DA release sites occur approximately every 4 µm along an axon [54,55]. Consequently, our verified experimental resolution of ~2 µm is well-positioned to resolve these dynamics, though resolution along the propagation direction remains coarser. The wide-field, micrometer-resolution readout of the PEM platform thus enables direct measurement of signaling phenomena predicted by the domain-overlap model [6].

In this model, the relevant functional variable is not just the peak concentration at a release site, but the lateral extent of dopamine spread and the subsequent crosstalk between signaling domains. By mapping the reach of the neurochemical signal and the total relative accumulation of signal over time, PEM can provide a functionally relevant representation of the integrated spatial distribution of extracellular DA, a level of detail lost in traditional single-point measurements.

### 4.4. Feasibility of Transitioning to Living Neuronal Networks

The current study establishes the analytical foundation needed to transition PEM from synthetic injection models to the complex environment of living cellular ensembles. To move toward real-time monitoring of neurochemical release from cells, a more refined quantitative correlation between integrated signal and delivered mass, and a robust and reproducible analytical framework must first be established, as detailed in the previous sections.

The adaptation of this platform for living cell studies presents technical challenges, most notably cell-mediated surface fouling and mechanical artifacts arising from cellular movement. We are currently developing a next-generation PEM architecture that implements a controlled-gap interfacial design to address these challenges. This configuration physically isolates the cell culture from the sensor surface to prevent biofouling and isolate cells from the applied potential, which could otherwise perturb cellular electrophysiology. Precise control of the separation distance (sub-micrometer) remains critical, allowing the system to capture the high local concentration of neurochemicals near the release site before it diffuses into the bulk solution and loses its spatial resolution. To maintain long-term cell viability, the next-generation system will incorporate a temperature-controlled perfusion chamber for maintaining physiological conditions at 37 °C and 5% CO_2_, while also facilitating the real-time introduction of stimulants or inhibitors.

The data-intensive nature of live-cell imaging will also require enhancements in algorithmic processing. We intend to leverage our established MATLAB-based analysis pipeline to implement automated spatiotemporal tracking algorithms that can quantify release events across the field of view.

## 5. Conclusions

In this work, we have successfully established plasmonic electrochemical microscopy (PEM) as a powerful, spatially resolved analytical platform for the real-time mapping of dopamine dynamics. By leveraging electrochemical polymerization of dopamine onto a functionalized gold interface, the PEM system achieved pixel-level quantification in bulk solutions, yielding a single-pixel limit of detection of approximately 14 pM. Furthermore, using a controlled delivery system, we demonstrated the platform’s unique capability to capture the localized release of micromolar dopamine in real time as a proof-of-concept for spatially resolved mass-responsive imaging. The platform can also visually decouple transient physical hydrodynamic forces from the persistent chemical signals of localized polydopamine film growth. This localized sensing mode functions as a spatially resolved mass integrator rather than a real-time concentration probe and provides spatial insight into the heterogeneous nature of surface-confined reactions. The resulting surface-bound ‘footprint’ provides a distinctive record of the cumulative spatial heterogeneity of dopamine release. This is biologically important because the total distribution and persistence of the neurotransmitter govern its physiological impact through volume transmission. By resolving these integrated spatial patterns, PEM has the potential to offer a deeper understanding of neurochemical distribution in complex environments, details that are lost in the single-point or spatially averaged measurements. Ultimately, this study establishes the solution-phase methodology and validation that will support meaningful interpretation of future cellular measurements. Building on this foundation, our next-generation PEM system, currently in development, will enable real-time mapping of dopamine release from living neuronal networks.

## Data Availability

Data used to generate figures for this manuscript can be accessed through Zenodo (https://doi.org/10.5281/zenodo.18676024).

## Supplementary Materials

The following supporting information can be downloaded at: https://www.mdpi.com/article/10.3390/bios16050284/s1, Figure S1: Experimental determination of spatial resolution using a gridded gold sensor; Figure S2: Experimental determination of spatial resolution using 10 mm polystyrene microbeads; Figure S3: Characterization of hydrodynamic disturbances via PBS injection; Video S1: Time-lapse sequence of background-subtracted PEM images capturing the localized dopamine delivery and polymerization.

## Abbreviations

The following abbreviations are used in this manuscript:

PEM	Plasmonic electrochemical microscopy
DA	Dopamine
EC-SPR	Electrochemical Surface Plasmon Resonance
FSCV	Fast Scan Cyclic Voltammetry
LOD	Limit of Detection
SPR	Surface Plasmon Resonance
SICM	Scanning Ion Conductance Microscopy
MEA	Microelectrode Array
