# Supplementary material for: Spatially Resolved Biosensing of Localized Dopamine Release via Its Electropolymerization Using Plasmonic Electrochemical Microscopy
Source: Biosensors (Basel). 2026 May 14;16(5):284. doi: 10.3390/bios16050284 (PMC13205039; doi:10.3390/bios16050284)
Supplement: Supplementary file 1 [file biosensors-16-00284-s001.zip › Supporting Information.pdf]

Supporting Information

Spatially Resolved Biosensing of Localized Dopamine Release via Its Electropolymerization Using Plasmonic Electrochemical Microscopy

Christian Martinez, Samuel Groysman, Madison Ngo, and Yixian Wang*

Department of Chemistry and Biochemistry, California State University, Los Angeles, Los Angeles. CA, United States

*Corresponding author

Yixian Wang; ywang184@calstatela.edu
Department of Chemistry and Biochemistry, California State University, Los Angeles, Los Angeles, CA, United States

Table of contents

S1 Determination of spatial resolution--3

Figure S1. Experimental determination of spatial resolution using a gridded gold sensor.

Figure S2. Experimental determination of spatial resolution using 10 µm polystyrene microbeads.

S2 Characterization of hydrodynamic disturbances via PBS injection-------------------------------------6

Figure S3. Characterization of hydrodynamic disturbances via PBS injection.

References--7

S1 Determination of spatial resolution.

To rigorously evaluate the spatial resolution of the PEM system, two complementary samples were utilized: a gridded gold sensor chip with etched micro-patterns and 10 μm microbeads deposited on bare gold sensors. By analyzing the intensity profiles at the grid edges and the signals from individual microbeads, we experimentally determined the system's analytical resolution.

Figure S1 illustrates the results from the gridded chip. We extracted intensity line profiles across both vertical and horizontal edges to measure the experimental transition width and compared it with the known physical edge width to isolate the optical broadening effect. Based on our previous characterization using atomic force microscopy, the physical edge width of these gridded chips is approximately 3 μm [1].

We first examined profiles perpendicular to the direction of surface plasmon propagation (left to right). Figure S1c–e show line profiles from three distinct locations. The shaded regions denote the transition width, which averaged 5.3 μm across 5–7 pixels. By subtracting the known 3 μm physical decay length, we calculated an experimental optical broadening of approximately 2.3 μm. This result confirms that the system can resolve features at the 2–3 μm scale in the lateral direction. Conversely, line profiles along the direction of plasmon propagation (Figure S1f and g) exhibit significant tailing and blurring extending tens of micrometers. This anisotropy is an inherent property of SPRM caused by the propagation length of the surface plasmon polaritons.

The microbead standard further confirms our conclusion regarding the system's spatial resolution. As shown in Figure S2a and b, the four identified microbeads generate parabolic tails due to the scattering of the surface plasmon wave, a characteristic pattern that is well-documented in the literature.[2,3] Crucially, as highlighted by both the plasmonic image (Figure S2B) and the line profile in Figure S2c, two microbeads in close proximity can be clearly isolated and distinguished from each other in the PEM view. This direct observation provides definitive proof of the system's ability to resolve individual micro-scale features, supporting its use for mapping local chemical activity in complex samples. Quantitative analysis of the line profiles supports this conclusion. The maximum width of the tails, measured perpendicular to the plasmon propagation direction, is approximately 13 pixels (12.2 μm). Subtracting the actual 10 μm size of the microbead yields an experimental optical broadening of approximately 2.2 μm. This value is consistent with the 2.3 μm lateral resolution derived from our gridded gold sensor tests and is sufficient to resolve individual dopamine signaling domains. The length of the tails, in the direction of plasmon propagation, is about 50 μm (Figure S2d).

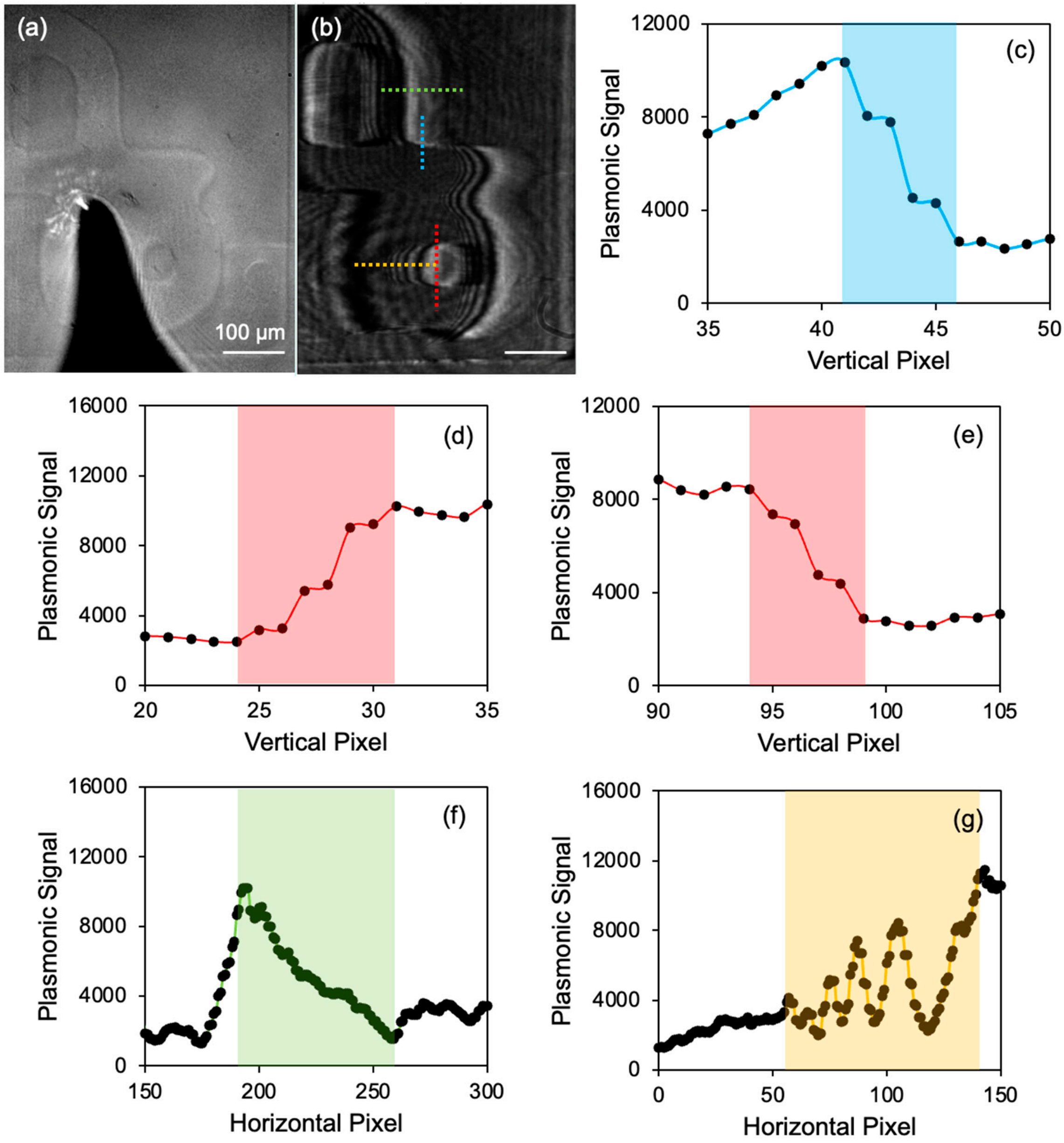

Figure S1. Experimental determination of spatial resolution using a gridded gold sensor.

(a) Bright-field and (b) plasmonic images of the same region on a gridded sensor chip. (c–g) Plasmonic intensity line profiles extracted along the corresponding colored dashed lines in (b). Shaded color blocks indicate the measured edge-transition width (decay length) across the grid boundaries. The gridded gold sensor chips were provided by Prof. Shaopeng Wang (Arizona State University) and fabricated via e-beam evaporation (PVD 75, Kurt J. Lesker, Jefferson Hills, PA, USA) by depositing a 2 nm Cr adhesion layer and a 47 nm Au layer on gridded glass coverslips (Bellco Glass, Inc., Vineland, NJ, USA). Coverslips were pre-cleaned with acetone in an ultrasonic bath for 10 min followed by a deionized water rinse. The black shadow in (a) is a micropipette positioned on top of the sensor.

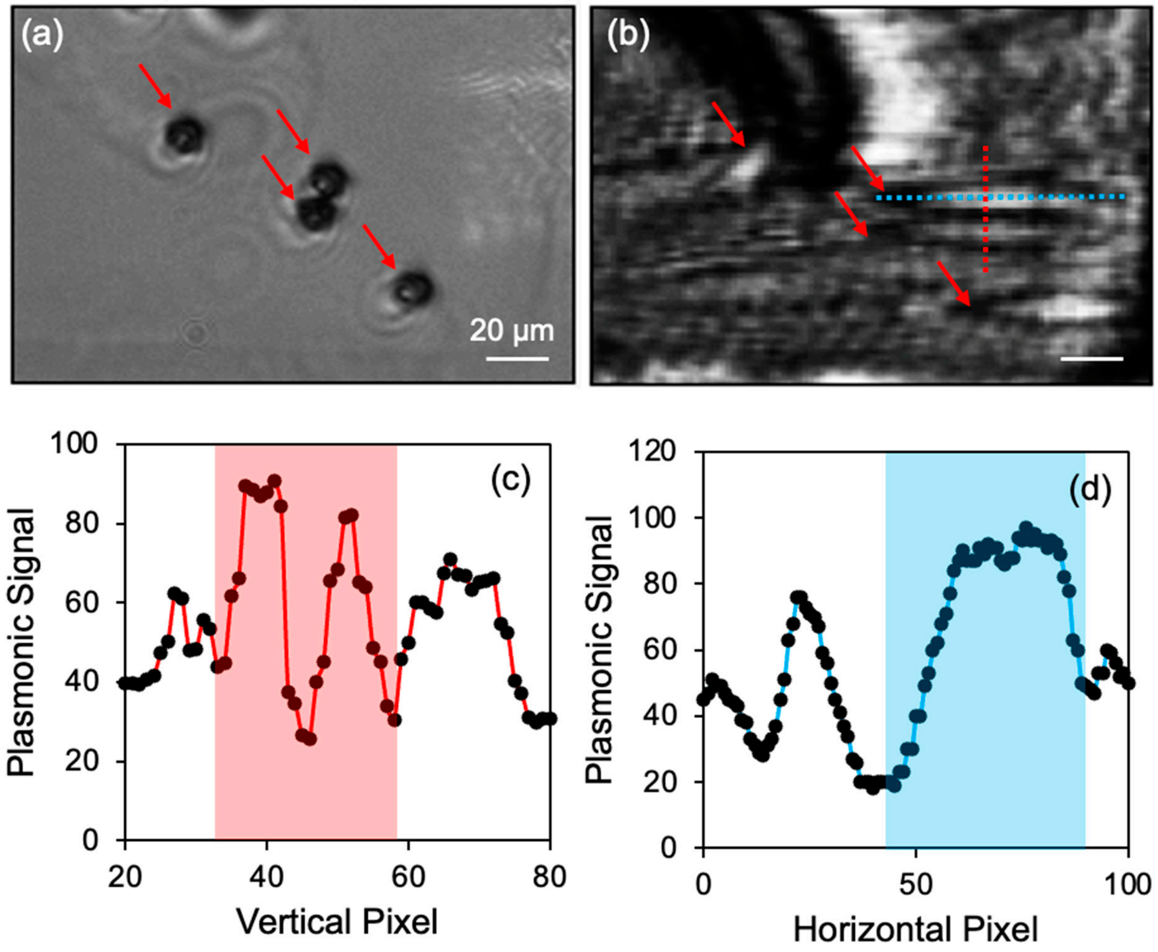

Figure S2. Experimental determination of spatial resolution using 10 µm polystyrene microbeads.

(a) Bright-field and (b) plasmonic images of the same region. Four microbeads are identified in both images with red arrows. Microbeads scattered the plasmonic wave and generated parabolic tails that start at their physical location. (c, d) Plasmonic intensity line profiles extracted along the corresponding colored dashed lines in (b). Shaded color blocks indicate the measured width (red) and length (blue) of the parabolic tails. (c) clearly demonstrates that the system can resolve two adjacent microbeads. Microbead standard (72986-5ML-F) was purchased from Sigma-Aldrich.

S2 Characterization of hydrodynamic disturbances via PBS injection

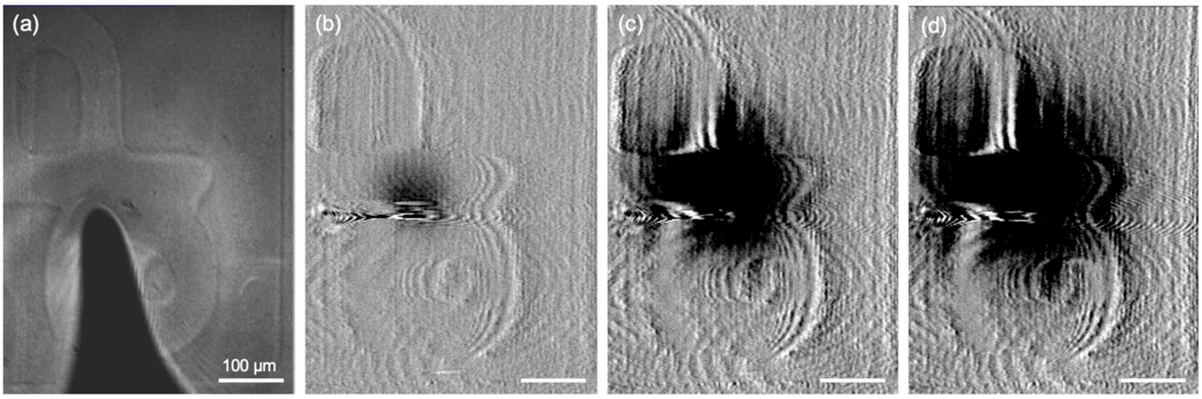

Figure S3. Characterization of hydrodynamic disturbances via PBS injection.

(a) Bright-field image indicating the spatial orientation of the micropipette relative to the gridded sensor. (b–d) Background-subtracted PEM snapshots recorded at 1 s, 2 s, and 3 s intervals following the initiation of a PBS injection. These frames capture the transient refractive index changes induced by local hydrodynamic pressure and flow, providing a baseline for distinguishing physical disturbances from persistent chemical signals.

References

1 S. Groysman, Y. Chen, A. Garcia, C. Martinez, K. Diego-Perez, M. Benavides, Y. Chen, Z. Wan, S. Wang, R. Liu, D. Wang, C. Liu, Y. Wang, Sensitive Imaging of Electroactive Species in Plasmonic Electrochemical Microscopy Enabled by Nanoconfinement, ACS Electrochem. 1 (2025) 974–986. https://doi.org/10.1021/acselectrochem.4c00227.

2 Y. Wang, X. Shan, H. Wang, S. Wang, N. Tao, Plasmonic Imaging of Surface Electrochemical Reactions of Single Gold Nanowires, J. Am. Chem. Soc. 139 (2017) 1376–1379. https://doi.org/10.1021/jacs.6b10693.

3 H. Yu, X. Shan, S. Wang, N. Tao, Achieving High Spatial Resolution Surface Plasmon Resonance Microscopy with Image Reconstruction, Anal. Chem. 89 (2017) 2704–2707. https://doi.org/10.1021/acs.analchem.6b05049.
